# Plagiarisms

**Published:** 1874-04-15

**Authors:** 


PLAGIARISMS.—From the criticisms of a correspondent, in the present number of THE EXAMINER, on the address of Dr. McArthur, and those which have appeared in the papers and journals concerning one by Dr. Palmer, of Michigan, we are inclined to think it would be a good, prudential move for our State medical societies to keep a sharp standing committee on plagiarisms, to which all addresses should be referred before publication.

